# Supplementary material for: A Rapid Head Organ Localization System Based on Clinically Realistic Images: A 3D Two Step Progressive Registration Method with CVH Anatomical Knowledge Mapping
Source: Bioengineering (Basel). 2024 Sep 1;11(9):891. doi: 10.3390/bioengineering11090891 (PMC11428975; doi:10.3390/bioengineering11090891)
Supplement: Supplementary file 1 [file bioengineering-11-00891-s001.zip › bioengineering-3096642-SI.pdf]

S.1 Preprocessing process of CVH dataset

S.1.1 CVH Labeling

CVH-2, as the second dataset, uses 24-bit true color tomographic images (slices) to record normal Asian human body structure, ie, Fig.S.1.A and Fig.S.1.B. And different tissues and organs were labeled slice by slice with different colors, and saved in different layers by Photoshop, thus establishing a complete CVH anatomical structure map database, ie, Fig.S.1.C and Fig.S.1.D.

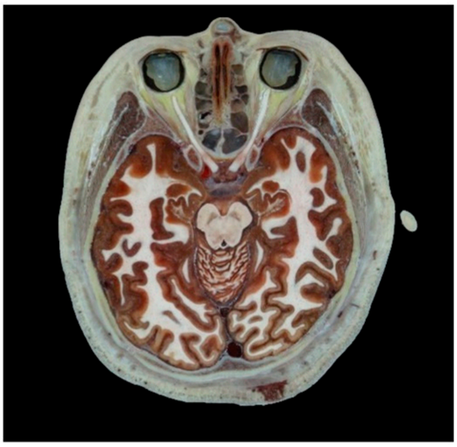

A. The 421st slice of Cross-Section

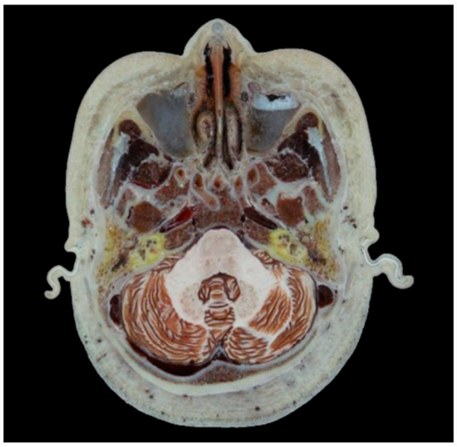

B. The 500st slice of Cross-Section

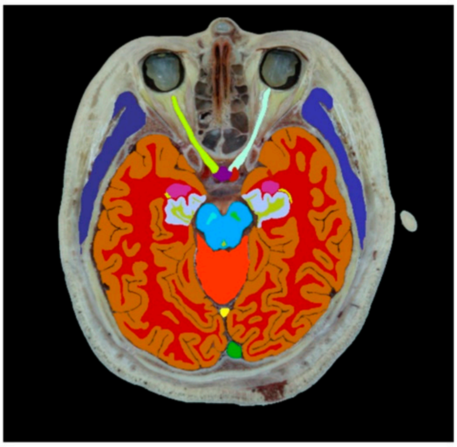

C. The 421st slice Covered with Organ labeling layer

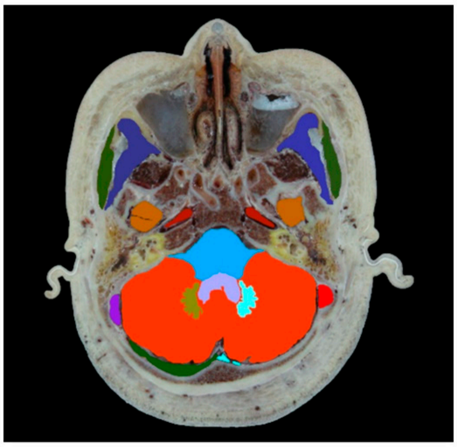

D. The 500st slice Covered with Organ labeling layer

Fig.S.1. Cross-sectional data and organ labeling of the CVH head region of 24-bit true color

At present, the head and neck region of CVH-2 has been annotated with 128 tissue parts from the parietal bone, frontal bone, dura mater, optic nerve, brainstem, arteries, median ligament of the thyroglossal bone to the oesophagus, which can satisfy the universal needs of ROI localization.

The above work complies with the regulations of the Chinese Ethics Committee.

S.1.2 Image compression

The original image of the head and neck of CVH-2 is shown in Fig.S.2, the resolution of each slice is 3872 × 2048, and the average file size of each image is 36MB, with a total of 1018 slices of sequential images, and the total amount of data is 1.62GB after PNG compression. Among them, the data spacing of the 1st~800th slices is 0.25mm, and the data spacing of the 801th~1018th slices is 0.5mm. Because of the huge amount of data and the inconsistent spacing of data slices, this paper has performed spatial size compression and normalization to adapt to the lightweight application on the client side.

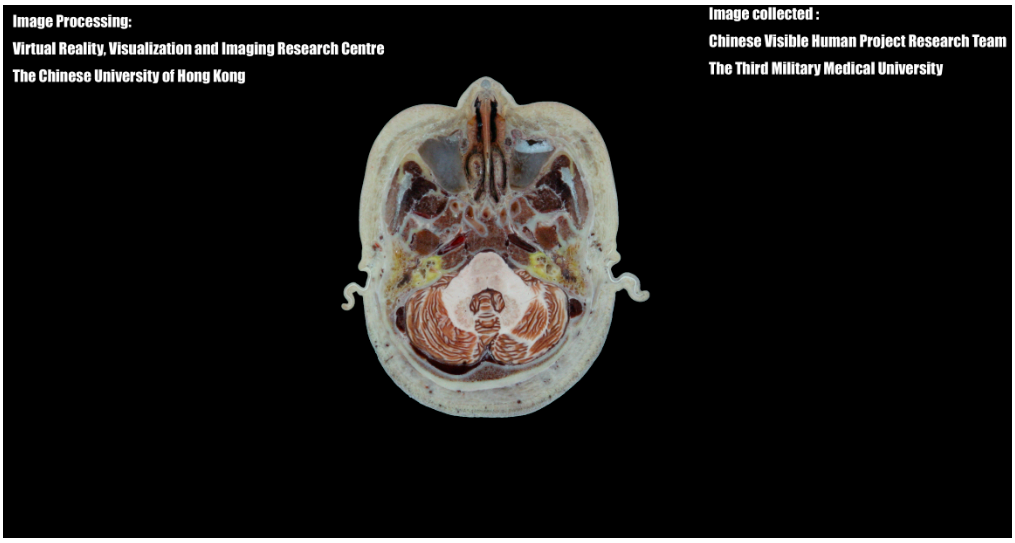

Fig.S.2. Raw CVH data slice

According to the characteristics of the original data of CVH head and the target requirements of this paper, the following preprocessing rules were designed:

Step 1, Set the data range of each slice of images to initially reduce the amount of data.

The data range interval of each slice of images is uniformly set to (1100,220)-(2600,1720), and only the data information needed for this study is retained to reduce the data volume. The resolution of head tomography data set per slice was reduced to 1500×1500.

Step 2, Standardized extraction of data slice intervals.

Setting 0001th~0800th slices to retain even layer data, and 0801th~1018th slices to retain all data, finally extract 619 slices of head tomography data.

Step 3, Shrink the resolution of each obtained slice into 256×256

Finally 619 shrunk slices of head tomography data were obtained, with a total data amount of 39.8 MB of lightweight dataset.

Step 4, the anatomical structure layers(organ labeling) overlaid on each slice operated in the same scale.

S.1.3 Normalization of visualization parameters

The raw tomographic color image sampling of the head of CVH-2 was set to have a *Pixel spacing* of 0.167mm in both x and y directions for each slice of the section, and a *Slice thickness* of 0.25mm for the first 800 slices and 0.5mm for the last 218 slices. Therefore, the original voxel spacing is 0.167mm×0.167mm×0.25mm for the first 800 slices and

0.167mm×0.167mm×0.5mm for the last 218 slices. According to the spatial dimension reduction requirement in Section S.1.2, the normalized formula for setting each layer of head CVH-2 data is:

$$\begin{bmatrix} Spacing\ X \\ Spacing\ Y \end{bmatrix} = \begin{bmatrix} ROI_w & \cdot 0.167 & \div SHK_w \\ ROI_h & \cdot 0.167 & \div SHK_h \end{bmatrix} \quad (S.1)$$

Where $[Spacing\ X,\ Spacing\ Y]^T$ denotes the pixel point spacing of each slice of CVH-2 after shrunk, $[ROI_w, ROI_h]^T$ denotes the resolution of the original size of the data range of each slice of images, and $[SHK_w, SHK_h]^T$ denotes the resolution of each slice after spatial size compression. After the normalized conversion process of Equation (S.1), the spacing of adjacent pixel points in the cross-section Spacing X × Spacing Y: 0.978515625mm × 0.978515625mm, i.e., the collective pixel spacing of the new CVH head true color tomography data is unified as follows: 0.978515625mm × 0.978515625mm × 0.5 mm.

S.1.4 Establishment of CVH anatomical knowledge database

The anatomical structures covered on each slice of images in CVH-2 are saved in layers according to the anatomical parts. Therefore, in this paper, all the layers of all PSD files corresponding to the head region are extracted, synchronized with the size change based on the processing of the original images according to Sections S.1.1-S.1.3, merged according to the same anatomical structure name of each organization (layer name), and saved in a folder named after the anatomical structure, so as to establish a knowledge database of gross anatomical structures, shown in Fig.S.3.

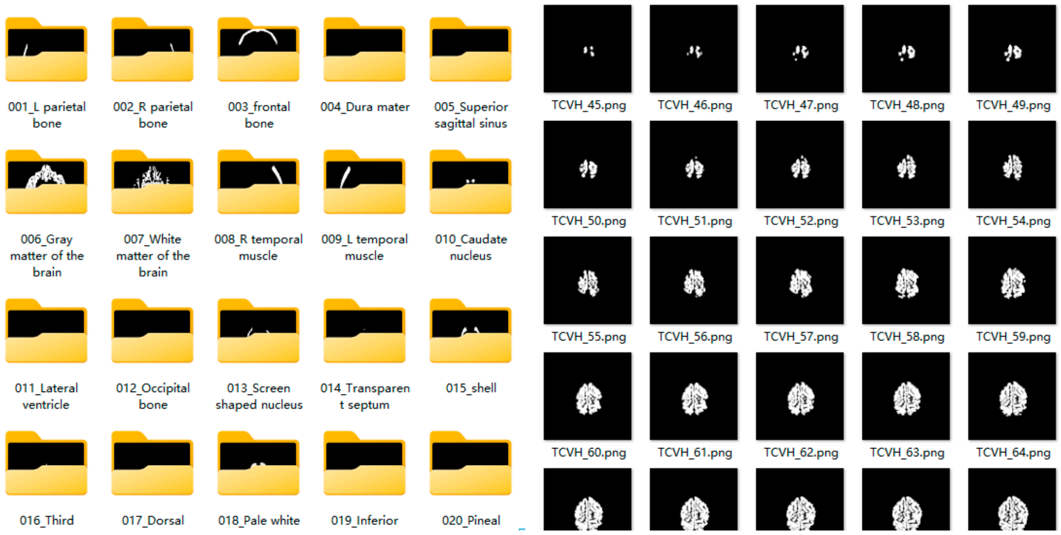

Fig.S.3. Classification and storage results of anatomical structures extracted from PSD layers

At present, the gross anatomical structures of the head and neck include 128 tissue or organization parts such as right and left parietal bones, frontal bones, dura mater, grey matter of the brain, white matter, pallidum, hippocampus, right and left optic nerves, pituitary gland, right and left dentate nuclei, right and left cephalic pinchers, parotid glands, spinal cord, etc., which satisfy the general needs of ROI localization.

S.2 Detailed Explanation of Homogeneous Transformation Matrices in TSPR Method

In the ICP algorithm, as the feature points of the CVH move towards the TMIs, the final R (rotation) and T (translation) are determined by continuously transforming R and T in Eq. (1), and then calculating the spatial distance differences between the two sets of feature points to find the optimal (minimum) solution. M_{1ij} (matrix1), M_{2ij} (matrix2), and M_{ij} (matrix) are 4x4 homogeneous matrices in Eq. (2), which are the matrix representations of R and T in Eq. (1).

R and T converted to matrix representation are as follows:

1. Rotation matrix R and translation vector T:
   - R is a 3x3 rotation matrix.
   - T is a 3x1 translation vector.
2. Constructing the homogeneous transformation matrix Mx_{ij}:
   - The homogeneous transformation matrix Mx_{ij} is a 4x4 matrix, which combines R and T together.

The form of the homogeneous transformation matrix Mx_{ij} is as follows:

$$Mx_{ij} = \begin{bmatrix} R_{11} & R_{12} & R_{13} & T_1 \\ R_{21} & R_{22} & R_{23} & T_2 \\ R_{31} & R_{32} & R_{33} & T_3 \\ 0 & 0 & 0 & 1 \end{bmatrix}$$

Where:

- R_{ij} are the elements of the rotation matrix R.
- T_i are the elements of the translation vector T.

M_{1ij} (matrix1) is the homogeneous matrix obtained when the CVH aligns with the TMIs during the first rigid registration (fixing spatial orientation);

M_{2ij} (matrix2) is the homogeneous matrix obtained when the CVH aligns with the TMIs during the second similarity registration (fixing spatial scale) based on the first transformation of the CVH. This step can be repeated xx times, denoted as M_{2ijX};

M_{ij} (matrix) is the homogeneous matrix from the initial position of the CVH to the end of the second registration. Therefore, it is calculated using Eq. (3).
